# Electroacupuncture on ST36 and GB39 Acupoints Inhibits Synovial Angiogenesis via Downregulating HIF-1*α*/VEGF Expression in a Rat Model of Adjuvant Arthritis

**DOI:** 10.1155/2019/5741931

**Published:** 2019-06-16

**Authors:** Jun Zhu, Chengguo Su, Yuzhou Chen, Xinyu Hao, Jianzhen Jiang

**Affiliations:** School of Acupuncture-Moxibustion and Tuina, The Third Affiliated Hospital, Chengdu University of Traditional Chinese Medicine, Chengdu 610075, China

## Abstract

*Introduction. *The hypoxia inducible factor-1*α* (HIF-1*α*) and vascular endothelial growth factor (VEGF) play a key role in synovial angiogenesis in rheumatoid arthritis (RA). Therefore, this study aimed to test the hypothesis that electroacupuncture (EA) may inhibit RA synovial angiogenesis via HIF-1*α*/VEGF expression.* Methods.* Sprague-Dawley rats were randomly distributed to 4 groups: control, adjuvant arthritis (AA), AA+electroacupuncture (AA+EA), and AA+sham EA groups. AA model was induced by injection of Freund's complete adjuvant in bilateral hind footpad. 3 days after injection, EA was delivered to the acupoints Zusanli (ST 36) and Xuanzhong (GB 39) once every two days for a total of 8 times in the AA+EA group, while sham EA treatment was applied in the AA+sham EA group. The arthritis score, paw volume, and H&E staining for each animal were measured. CD34 expression in synovial tissue of ankle joint was observed by immunohistochemistry. HIF-1*α* and VEGF mRNA and protein levels in synovial tissue were determined by real-time quantitative PCR and Western blot, respectively.* Results.* Compared with rats in AA group, EA stimulation significantly decreased arthritis scores, paw volume, and pathological damage of synovial tissues. Moreover, EA markedly suppressed the synovial angiogenesis of AA rats, as evidenced by reduced CD34 positive expression. Furthermore, EA significantly reduced HIF-1*α* and VEGF mRNA and protein levels in synovial of AA rats. Finally, the CD34 expression in synovial tissue was positively correlated with HIF-1*α* and VEGF protein levels.* Conclusion*. EA on ST36 and GB39 acupoints can effectively inhibit synovial angiogenesis in the AA rat model via downregulating HIF-1*α*/VEGF expression.

## 1. Introduction

Rheumatoid arthritis (RA) is a systematic and autoimmune disorder and synovial angiogenesis plays a critical role in its development process [1, 2]. Previous studies have shown that angiogenesis is the development of abnormal capillaries that enter the synovial tissue and cartilage from existing blood vessels in the presence of synovial inflammation and exacerbates inflammatory response of synovium, resulting in hypoxia of the synovial and destruction of cartilage [3, 4]. In addition, evidence indicates that hypoxia inducible factor-1*α* (HIF-1*α*) and vascular endothelial growth factor (VEGF) may play a key signaling-mediated role in this process [5]. HIF-1*α* is an important cellular hypoxic factor and a typical proangiogenic factor [6]. In RA synovial tissue, inflammation contributes to dramatically increased HIF-1*α* expression, which promotes synovial cells to be more tolerant of hypoxic challenge [7]. VEGF, a downstream target of HIF-1*α*, regulates angiogenesis of RA. It has been demonstrated that HIF-1*α* induces synovial cells to secrete VEGF and stimulates synovial angiogenesis, which in turn leads to aggravation of RA [8]. Simultaneously, synovial angiogenesis also feeds the expression of HIF-1*α* and VEGF in synovial tissue [7].

Electroacupuncture (EA) could accelerate middle cerebral artery occlusion-induced angiogenesis and enhance recovery of neurological function by upregulating HIF-1*α* expression [9, 10]. EA has good clinical efficacy in the treatment of RA and can reduce the expression of VEGF [11, 12]. Animal experiments also confirmed that EA can regulate the immune network and inhibit arthritis in RA animal model [13]. Some studies have revealed that EA on Xuanzhong (GB39, located at four-fifths of the distance from the lateral knee to the lateral malleolus of the tibiofibula) and Zusanli (ST36, distal to the head of the tibia in a depression between the muscles of the cranial tibia and the long digital extensor) have a potential effect on RA and can effectively improve symptoms of RA, which has been selected in many clinical studies [14, 15]. Our previous investigation indicates that EA on GB39 and ST36 acupoints attenuates the severity of RA in rats via upregulating vasoactive intestinal peptide receptor 1 and reestablished the T helper 17/regulatory T cell balance [16]. However, the involved mechanisms are poorly understood. In this study, we aimed to evaluate the effects of stimulating GB39 and ST36 acupoints on HIF-1*α*/VEGF expression by a AA rat model.

## 2. Materials and Methods

### 2.1. Ethics Statement

This study was approved by the Scientific Investigation Board of the Chengdu University of Traditional Chinese Medicine, Chengdu, China (approval number: CUTCM-2018-05). All animal experiments were designed according to the principles of the 3Rs (Replacement, Reduction and Refinement) and were carried out in accordance with the National Institutes of Health Guide for the Care and Use of Laboratory Animals [17].

### 2.2. Animals

Forty male Sprague-Dawley rats (6 weeks of age, 180 g) were purchased from Chengdu Dashuo Laboratory Animal Co. Ltd. (Chengdu, China) and dewelled in a pathogen-free environment with two animals per cage. A 12 h light-dark cycle was maintained and rats had free access to standard rodent chow and water.

### 2.3. Experimental Design and Induction of AA

**Figure 1:**
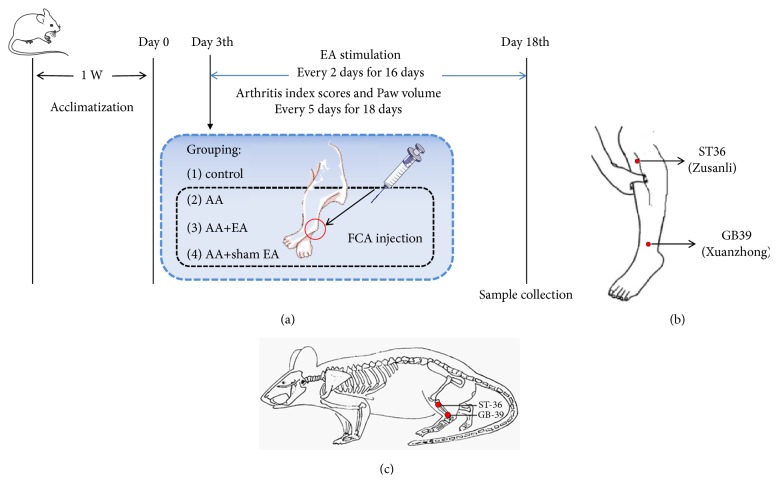
Study design of the experimental procedure. Rats were randomly grouped into control, adjuvant arthritis (AA), AA+electroacupuncture (AA+EA), and AA+sham EA groups with 10 rats in each. Freund's complete adjuvant was injected in bilateral hind footpad to establish rat model of AA after anesthesia, rats in control group were injected with saline. 3 days after the immunization, rats in AA+EA group were performed EA on acupoints ST 36 and GB 39, and rats in AA+sham EA group were subjected to an electrical stimulation at a nonacupoint once two days for a total of 8 times. Arthritis scores and paw volume for each rat were determined every 5 days for 4 times after the injection. Rats were sacrificed after 18 days and the bilateral ankles were sampled for further examination. The left ankle joint was used for H&E staining and immunohistochemistry analysis, and the right ankle joint synovium was used for PCR and Western blot analysis.

Rats were randomly grouped into control, adjuvant arthritis (AA), AA+electroacupuncture (AA+EA), and AA+sham EA groups with 10 rats in each. The rat model of AA was established as described previously [18]. After anesthesia with an intraperitoneal injection of pentobarbital (35 mg/kg), rats in control group were injected with saline and the others were injected with 0.1 ml Freund's complete adjuvant (FCA, Product Number: F5881, Sigma, St. Louis, Missouri, USA) in bilateral hind footpad. 3 days after the immunization, the degree of swelling of the bilateral hind limb ankle joints was observed and scored according to previous research [19]. More than 3 points indicated that AA model was successfully established. Additionally, rats in AA+EA group were performed EA on acupoints ST 36 and GB 39, and rats in AA+sham EA group were subjected to an electrical stimulation at a nonacupoint (3 mm lateral to the side of GB39 and ST36) once two days for a total of 8 times. Arthritis scores and paw volume for each rat were determined every 5 days for 4 times after the injection. Rats were sacrificed after 18 days and the bilateral ankles were sampled for further examination. The left ankle joint was used for H&E staining and immunohistochemistry analysis, and the right ankle joint synovium was used for PCR and Western blot analysis. The research scheme designed in this experiment is presented in Figure 1.

### 2.4. EA Treatment

Three days after FCA injection, EA was performed as described previously [16]. In brief, two stainless steel needles (0.25×25 mm; Suzhou Medical Products Factory Co., Ltd. Suzhou, China) were inserted perpendicularly at GB39 (3 mm depth) and ST36 (6 mm depth). The needles were further connected to an electronic acupuncture treatment instrument (Hwato SDZ-II, Suzhou Medical Products Factory Co., Ltd., Suzhou, China). Finally, a constant electrical stimulus (2 Hz, 0.2 ms pulse width) was performed for a continuous 15 min. The intensity (6-7 mA) of the acupuncture current was adjusted according to the appearance of muscle contractions around the acupuncture points. Rats in AA+sham EA group received the same electrical stimulation procedures at 3 mm lateral to the side of GB39 and ST36. All stimulation procedures were performed every 2 days for 16 days.

### 2.5. Arthritis Index Scoring and Paw Volume

The arthritis index score was evaluated three days after the FCA injection. Two individuals independently evaluated the arthritis index score of each rat, every 5 days for 18 days. The arthritis index score was assessed using a scoring system with five grades [19]: 0, no evidence of erythema or swelling; 1, erythema and slight swelling confined to the mid-foot (tarsal) or ankle joint; 2, erythema and mild swelling extending from the ankle to the mid-foot; 3, erythema and moderate swelling extending from the ankle to the metatarsal joints; grade 4, erythema and severe swelling involving the ankle, foot and digits. The mean values of the arthritis index scores were calculated and plotted at each time point.

The mean paw volume of bilateral hind claws of the rats was measured by volume drainage method using a plethysmograph apparatus (YLS-7A, Yiyan Sci Ltd., Jinan, China) every 5 days for 18 days.

### 2.6. H&E Staining

The isolated ankle joint was washed in cold phosphate-buffered saline (PBS, cat. no. G0002; Solarbio, Beijing, China), fixed with 4% paraformaldehyde, and embedded in paraffin wax. After sectioning, 4 *μ*m-thick sections were deparaffinaged in xylene followed by rehydration through an ethanol gradient. H&E staining was performed prior to histopathological examination by a blinded assessor (Microscope: NIKON Eclipse ci, imaging system: NIKON digital sight DS-FI2, Microscope multiple: 100×). The stained sections were quantified by 2 investigators who were blinded to the treatments according to previous literature report [15].

### 2.7. Immunohistochemistry

Immunohistochemistry was performed by DAKO Envision+ Reagent (DakoCytomation, Carpinteria, CA, USA) as previously described [20]. Briefly, paraffin sections (4-*μ*m thick) were deparaffinized with xylene, rehydrated, and microwave antigen retrieval by containing 10 mM of sodium citrate (pH 6.0). Sections were incubated with rabbit anti-CD34 primary antibody (cat. no. ab81289; Abcam, Cambridge, UK; in dilution 1:200) at 4°C for 12h. The sections were subsequently incubated with goat anti-rabbit antibody conjugated to horseradish peroxidase (cat. no. 70-GAR007; MultiSciences, Hangzhou, China; in dilution 1:200) at 26°C for 50 min, followed by 3,3′-diaminobenzidine (DAB) staining. The CD34 expression of synovial angiogenesis was recorded under a 200-fold light microscope (Nikon Eclipse C1, Tokyo, Japan). 3 fields of each sample were randomly observed and the mean optical density of positive brownish yellow was calculated using Image-Pro Plus software version 6.0 (Media cybernetics, Inc.). Negative controls were included by omitting the primary antibody, and a known positive control was included with each batch. The staining results were evaluated by 2 experienced pathologists without knowing the group allocations.

### 2.8. RNA Isolation and Real-Time Quantitative PCR

Total RNA from rat ankle synovial tissue was extracted using an RNA extraction kit (cat. no. G3013; Servicebio, Wuhan, China) according to the manufacturer's instructions. cDNA was subsequently synthesised from total RNA using the cDNA Synthesis Kit (cat. no. #K1622; Thermo, Mannheim, Germany). Real-time quantitative PCR for HIF-1*α* and VEGF was performed with a SYBR-green detection kit (cat. no. 04913914001; Roche, Basel, Switzerland) on a ABI Stepone plus Real-time PCR System (Applied Biosystems, Foster City, California, USA). Glyceraldehyde-3-phosphate dehydrogenase (GAPDH) was used as reference gene for normalization of different transcript values. The thermocycling conditions were as follows: initial denaturation at 95°C for 10 min, followed by 40 cycles of denaturation at 95°C for 15 s, annealing at 60°C for 60 s, elongation at 72°C for 30 s, and a final extension step at 72°C for 5 min. The primers sequence for HIF-1*α* and VEGF was listed in Table 1. All PCR assays were performed in triplicate. The relative mRNA levels were calculated according to the 2^−ΔΔCt^ method [21].

### 2.9. Western Blot

The synovial tissue was washed 2-3 times with cold PBS and was cut into small pieces and placed in the homogenizer. Add then the cold whole cell lysis buffer (cat. no. G2002; Servicebio, Wuhan, China) of 10 times tissue volume was added and thoroughly homogenized on ice. After that the homogenate was transferred to 1.5 ml centrifugal tube and bathed in ice for 30 mins, during which the cells were blown repeatedly with a pipette to ensure complete cell lysis. Finally, the supernatant was collected by centrifugation of 14,000 x g for 5 min at 4°C. Protein concentration in the supernatant was standardized with a protein concentration assay kit (cat. no. G2026; Servicebio, Wuhan, China). A total of 40 *μ*g protein per lane was separated using 12% SDS-PAGE and transferred to polyvinylidene difluoride membranes. The membranes were blocked with 5% bull serum albumin (cat. no. G2013; Servicebio, Wuhan, China) for 2 h at 25°C, followed by incubation with HIF-1*α* (cat. no. ab51608; Abcam, Cambridge, UK; in dilution 1:1,000), VEGF (cat. no. ab32152; Abcam, Cambridge, UK; in dilution 1:1,000), and GAPDH (cat. no. GB12002; Servicebio, Wuhan, China; in dilution 1:1,000) overnight at 4°C. The next day, membranes were incubated with horseradish peroxidase-conjugated goat anti-rabbit immunoglobulin G secondary antibodies (cat. no. GB23303; Servicebio, Wuhan, China; in dilution 1:1,000) for 2 h at 25°C. Finally, protein bands were developed using a ChemiDoc™ XRS Imaging system (Bio-Rad Laboratories, Inc., Hercules, CA, USA). The bands of interest were calculated and normalized to GAPDH using the Image-Pro Plus software version 6.0 (Media cybernetics, Inc.).

### 2.10. Statistical Analysis

All data were expressed as means±SD. Multigroup comparison was evaluated using ANOVA test followed by Tukey multiple comparison tests. ANOVA of repeated measurement data was used to measure arthritis index scores and paw volume. Spearman bivariate correlation was used to determine the relation between the CD34 expression and the protein expression of HIF-1*α* and VEGF. All statistical analyses were performed by SPSS 20.0 (SPSS, Chicago, IL, USA). P<0.05 was considered to be statistically significant.

## 3. Results

### 3.1. EA Reduces Arthritis Index Scores and Paw Volume in AA Rats

**Figure 2:**
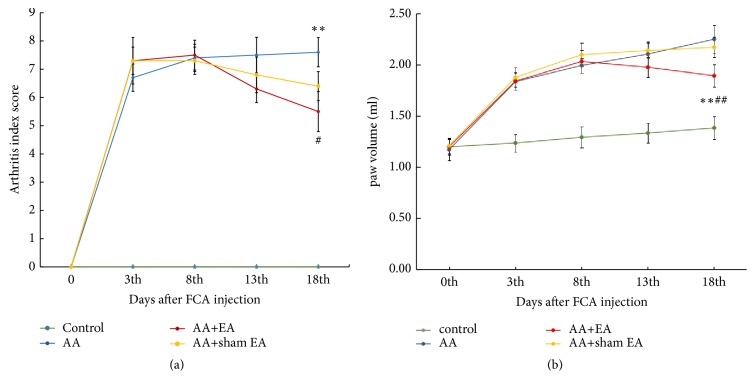
Arthritis index score (a) and paw volume (b). Measurements of arthritis index scores and paw volume over a 18-day period in 40 rats injected with saline (control, n=10) or Freund's complete adjuvant to establish a rat model of adjuvant arthritis (AA, n=30). AA rats were treated with electroacupuncture (EA) alone (AA+EA, n=10) or sham EA (AA+sham EA, n=10). Data are means±SD.  ^*∗*^P <0.05;  ^*∗∗*^P <0.01, compared with the AA group. ^#^P <0.05; ^##^P <0.01, compared with control group.

The arthritis index scores are shown in Figure 2(a). The scores in AA and AA+sham EA groups significantly increased (*P* > 0.05) and did not reduce over time compared with the control group. However, compared with AA and AA+sham EA groups, EA treatment can effectively reduce the arthritis index scores of AA rats (*P* < 0.01).

The paw volume is shown in Figure 2(b). The scores in AA and AA+sham EA groups significantly increased compared with the control group (*P* > 0.05). However, compared with AA and AA+sham EA groups, EA treatment can effectively reduce the paw volume of AA rats (*P* < 0.01).

### 3.2. EA Reduces H&E Staining Score in AA Rats

**Figure 3:**
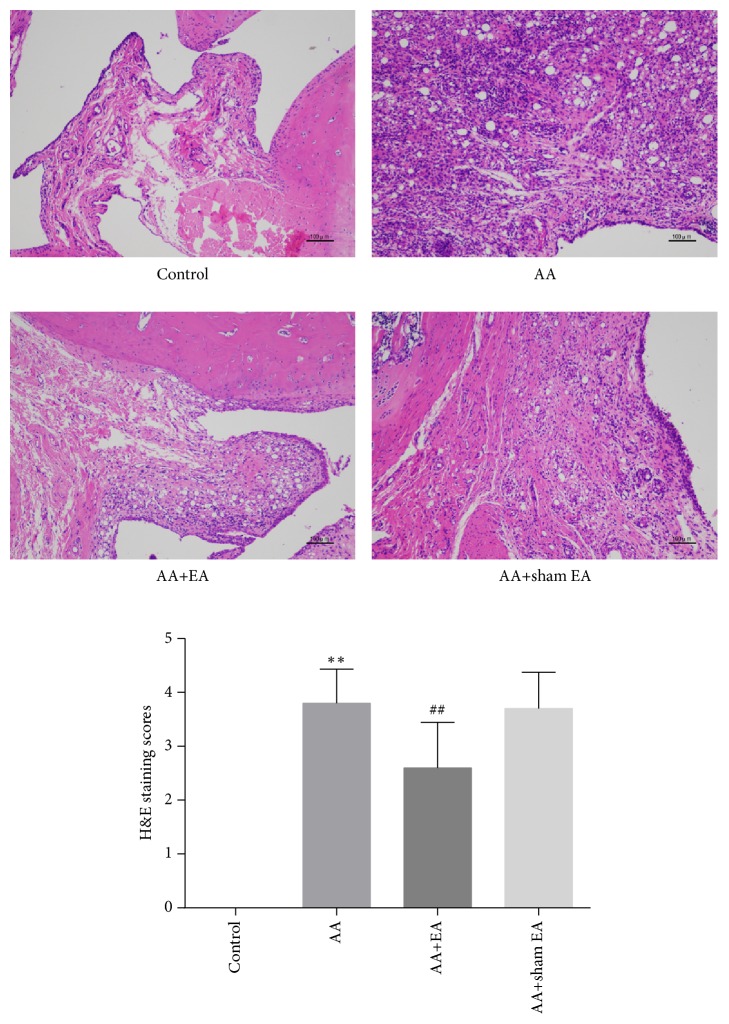
Inflammation in synovial tissue of the ankle joint. Representative H&E-stained sections from the* control* group, demonstrating no evidence of inflammation in the ankle joint, the* AA* group and the* AA+sham EA* group illustrating massive inflammatory infiltration, synovial proliferation, and angiogenesis in the swollen joints, and the* AA+EA* group, demonstrating less inflammatory cell infiltration, synovial proliferation and angiogenesis in the ankle joint (n=10) (×100). Data are means±SD.  ^*∗*^P <0.05;  ^*∗∗*^P <0.01, compared with the AA group. ^#^P <0.05; ^##^P <0.01, compared with control group.

The result of H&E staining showed that, compared with control group, significant inflammatory infiltration and synovial proliferation were observed in AA and AA+sham EA groups (*P* < 0.05) (Figure 3), and H&E staining scores show no significant difference between AA and AA+sham EA groups (*P* > 0.05), while compared with AA group, EA on ST36 and GB39 effectively reduced H&E staining scores in AA rats (*P* < 0.05). However, compared with AA+sham EA group, electroacupuncture intervention can significantly reduce H&E staining score (*P* < 0.05).

### 3.3. EA Inhibits CD34 Expression in Synovial Angiogenesis of AA Rats

**Figure 4:**
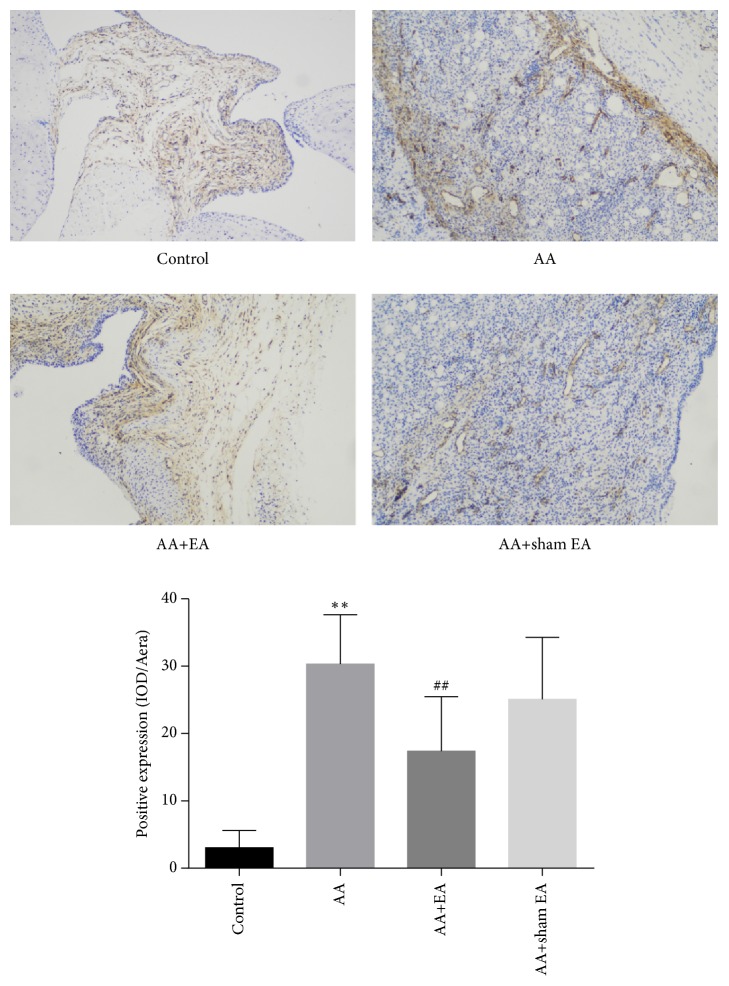
Measurement of synovial CD34 expression by immunohistochemistry. H&E-stained sections from the* control* group, demonstrating CD34 was only expressed in a small amount in the ankle joint, the* AA* group and the* AA+sham EA* group illustrating a very large number of CD34 expression in the swollen joints, and the* AA+EA* group, demonstrating less number of CD34 expression in the ankle joint. Mean inflammation score (+SD) on a 3-point scale. Compared with control group,  ^*∗*^P < 0.05 and  ^*∗∗*^P < 0.01; compared with AA group, ^#^P < 0.05 and ^##^P <0.01, n=10 (n=10) (×200).

CD34 is a specific marker of synovial angiogenesis [22]. To identify the effect of EA on CD34 protein expression of ankle synovial tissue, immunohistochemistry was conducted. The results showed that CD34 expression was significantly elevated in the AA group compared with the control group (*P* < 0.01, Figure 4). The expression of CD34 in AA+EA group was significantly lower than that in the AA and AA+sham EA groups (*P* <0.01). However, compared with AA group, sham electroacupuncture stimulation had no effect on the expression level of CD34 (*P* > 0.05). These results indicated that EA on ST36 and GB39 significantly inhibited the expression of CD34 in synovial tissue.

### 3.4. EA Reduced the mRNA Expression of HIF-1*α* and VEGF in Synovial Tissue

**Figure 5:**
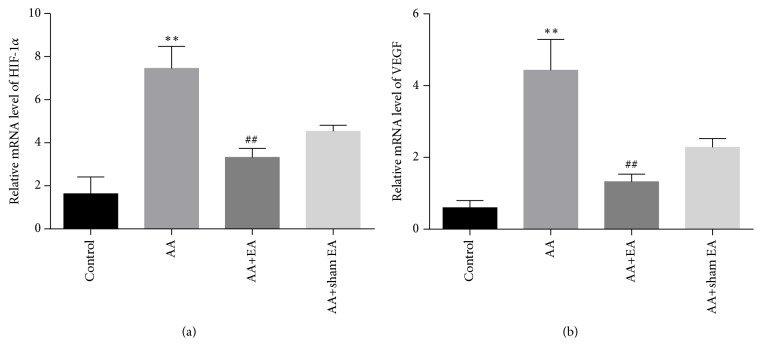
HIF-1*α* and VEGF mRNA expression in synovial tissue. Measurement of synovial HIF-1*α* and VEGF mRNA abundance by real-time quantitative-PCR analysis. (a) Summary of EA on expression of HIF-1*α* mRNA. (b) Summary of EA on expression of VEGF mRNA. Data are shown as mean ± SD, n=10. Compared with control group,  ^*∗*^P < 0.05 and  ^*∗∗*^P < 0.01; compared with AA group, ^#^P < 0.05 and ^##^P <0.01 (n=10).

The HIF-1*α* and VEGF mRNA expression in synovial tissue was detected by real-time quantitative PCR. The results showed that the expression of HIF-1*α* (*P* < 0.01, Figure 5(a)) and VEGF (*P* < 0.01, Figure 5(b)) mRNA in AA group was significantly higher than that in the control group. Sham electroacupuncture stimulation had no effect on the HIF-1*α* and VEGF mRNA levels (*P* > 0.05). However, the expression of HIF-1*α* and VEGF mRNA in AA+EA group was markedly decreased than that in AA group. Compared with AA+sham EA group, electroacupuncture intervention can significantly reduce the mRNA expression of HIF-1*α* and VEGF (*P* < 0.01). Therefore, the above results demonstrated that the EA on ST36 and GB39 significantly reduced the mRNA expression of HIF-1*α* and VEGF in synovial tissue.

### 3.5. EA Reduces the Protein Expression of HIF-1*α* and VEGF in Synovial Tissue

**Figure 6:**
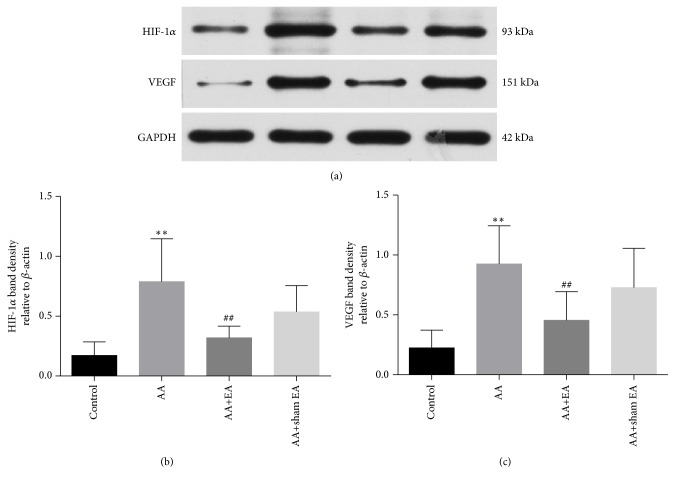
HIF-1*α* and VEGF protein expression in synovial tissue. (a) Western blot results showing expression levels of HIF-1*α* and VEGF protein. (b, c) Summary of EA on expression of HIF-1*α* and VEGF protein. Data are shown as mean ± SD, n=10. Compared with control group,  ^*∗*^P < 0.05 and  ^*∗∗*^P < 0.01; compared with AA group, ^#^P < 0.05 and ^##^P <0.01 (n=10).

The results of western blotting showed that HIF-1*α* (*P* < 0.01, Figures 6(a) and 6(b)) and VEGF (*P* < 0.01, Figures 6(a) and 6(c)) protein expression in the AA group was significantly higher than that in the control group. Compared with AA group, EA treatment on ST36 and GB39 strikingly inhibited HIF-1*α* and VEGF levels. Compared with AA+sham EA group, electroacupuncture intervention can significantly reduce the protein expression of HIF-1*α* and VEGF (*P* < 0.01). However, there was no significant different in the protein levels of HIF-1*α* and VEGF between AA+ sham EA and AA group (*P* > 0.05).

### 3.6. Correlation between CD34 Expression and HIF-1*α*/VEGF Protein Expression in Synovial Tissue

**Figure 7:**
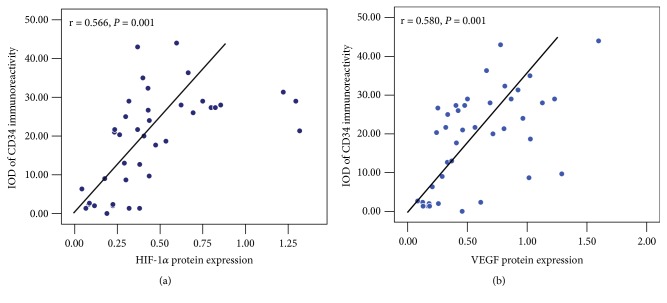
Relationship between CD34 immunostaining intensity and the protein expression of HIF-1*α* (a) and VEGF (b) in synovial tissue in rats with adjuvant-induced arthritis, n = 40.

To determine the correlation between CD34 and the protein expression of HIF-1*α* and VEGF in synovial tissue, spearman bivariate correlation analysis was conducted. As shown in Figure 7, CD34 expression intensity was significantly positively correlated with HIF-1*α* (r = 0.566,* P* < 0.01, Figure 7(a)) and VEGF (r = 0.580,* P* < 0.01, Figure 7(b)) protein expression. These data collectively suggested that synovial angiogenesis marker CD34 was positively related to the expression of HIF-1*α* and VEGF.

## 4. Discussion

EA stimulation can effectively control the progression of RA patients, and the related anti-inflammatory mechanism has also been explored [12, 13]. Low-frequency EA can inhibit joint inflammation in AA model through upregulating *α*2- and *β*-adrenoceptors and downregulating transient receptor potential vanilloid 1 and other signaling molecules, such as, NF-*κ*Β, TNF-*α*, IL-1*β*, IL-6, and IL-8 [23–25]. Furthermore, synovial angiogenesis is another typical feature of the development of RA [26]. ST36 is a common acupoint in electroacupuncture intervention for inflammation and immune regulation [27]. GB39 is an acupoint near the ankle joint, which can significantly inhibit local arthritis. In the theory of meridians and collaterals of traditional Chinese medicine, it has a significant beneficial effect and can markedly inhibit the progress of RA [28]. The combination of these two acupoints has a more significant effect on inhibiting arthritis, and it is also a classical acupoint pairing commonly used in clinical electroacupuncture treatment of RA patients [12, 14, 28]. We, therefore, synchronously stimulated both ST36 and GB39 acupoints to observe its therapeutic effect on AA rat model. EA on ST36 and GB39 significantly reduced the severity of RA animals, as evidenced by reduced paw volumes, arthritis scores, and inflammatory scores [15, 16]. In this study, the results of H&E stain consistently demonstrated that EA on ST36 and GB39 restrained the inflammatory responses and synovial proliferation in AA rats.

Clinical researches have shown that the effects of acupuncture or electroacupuncture intervention at acupoints are significantly better than that of sham acupuncture intervention at nonacupoints near acupoints [28–30]. In the animal experiment of anti-inflammatory effect of electroacupuncture, the pretreatment of Hegu acupoint can improve the survival rate of rats with lethal endotoxemia, but the same parameters of electroacupuncture intervention at nonacupoints (a set of nonacupoints located on the ulna side of the metacarpus served as controls) had no similar effect [31]. These results also suggest that the anti-inflammatory effect of electroacupuncture on acupoints is significantly better than that of nonacupoints. Our results also confirmed that sham electroacupuncture stimulation had no effect on arthritis scores, pathological lesion of synovial joint, vascular hyperplasia, and gene or protein expression of HIF-1*α* and VEGF, compared with AA group, which were consistent with previous reports.

CD34 is a specific marker of angiogenesis [32]. However, there is little literature on the association between electroacupuncture and CD34 expression. Additionally, HIF-1*α* and VEGF play a crucial role in the progression of RA disease, directly leading to the formation of synovial angiogenesis [33]. Inflammatory cytokines, such as TNF-*α*, IL-17, and IL-33 [34, 35], and local hypoxia in joints can cause exponential growth of HIF-1*α* in synovial tissue and stimulate the production and secretion of VEGF [36]. As a downstream signaling molecule of HIF-1*α*, VEGF is a special cytokine that acts directly on vascular endothelium of the synovium, promotes the formation of synovial angiogenesis, and increases vascular permeability and inflammatory cytokines production [37–39]. In the synovial fluid and peripheral serum of RA patients, HIF-1*α* and VEGF are detected to be highly expressed and closely related to the activity of the disease [40]. The present study also confirmed an accumulation of HIF-1*α* and VEGF protein and gene expression in a rat model of adjuvant arthritis. However, EA on ST36 and GB39 significantly reduced the mRNA and protein expressions of HIF-1*α* and VEGF in the synovial tissue of AA rats. Furthermore, the expression of CD34 in synovial tissue is positively correlated with HIF-1*α* and VEGF levels [41]. We further found that CD34 protein expression was positively correlated with HIF-1*α* and VEGF levels by a mathematical correlation analysis.

In conclusion, the present study investigated the inhibition of angiogenesis of EA on ST36 and GB39 on FCA-induced AA rat model* in vivo*. The results demonstrated that EA on ST36 and GB39 acupoints effectively inhibited the formation of synovial angiogenesis via downregulating HIF-1*α*/VEGF expression. However, the effectiveness of EA on ST36 and GB39 acupoints needs further clinical verification in AA patients, and more* in vitro* and* in vivo* studies are required to elucidate its underlying mechanisms.

## Figures and Tables

**Table 1:** Primers used for real-time quantitative PCR in this study.

Gene	GenBank accession number	Primers sequence (5′-3′)
GAPDH	NM_017008.4	
	Forward	CTGGAGAAACCTGCCAAGTATG
	Reverse	GGTGGAAGAATGGGAGTTGCT
HIF-1*α*	NM_024359.1	
	Forward	ACCGTGCCCCTACTATGTCG
	Reverse	GCCTTGTATGGGAGCATTAACTT
VEGF	NM_001110333.2	
	Forward	GTTCAGAGCGGAGAAAGCATT
	Reverse	CTTGCAACGCGAGTCTGTGT

## Data Availability

The data used to support the findings of this study are available from the corresponding author upon request.
